# The pursuit of approaches to federate data to accelerate Alzheimer’s disease and related dementia research: GAAIN, DPUK, and ADDI

**DOI:** 10.3389/fninf.2023.1175689

**Published:** 2023-05-25

**Authors:** Arthur W. Toga, Mukta Phatak, Ioannis Pappas, Simon Thompson, Caitlin P. McHugh, Matthew H. S. Clement, Sarah Bauermeister, Tetsuyuki Maruyama, John Gallacher

**Affiliations:** ^1^Laboratory of Neuro Imaging, USC Stevens Neuroimaging and Informatics Institute, Keck School of Medicine of USC, University of Southern California, Los Angeles, CA, United States; ^2^Alzheimer’s Disease Data Initiative, Kirkland, WA, United States; ^3^Department of Psychiatry, Warneford Hospital, University of Oxford, Oxford, United Kingdom

**Keywords:** federated data access, Alzheimer’s disease and neurodegeneration, data sharing, dementia–Alzheimer’s disease, remote data

## Abstract

There is common consensus that data sharing accelerates science. Data sharing enhances the utility of data and promotes the creation and competition of scientific ideas. Within the Alzheimer’s disease and related dementias (ADRD) community, data types and modalities are spread across many organizations, geographies, and governance structures. The ADRD community is not alone in facing these challenges, however, the problem is even more difficult because of the need to share complex biomarker data from centers around the world. Heavy-handed data sharing mandates have, to date, been met with limited success and often outright resistance. Interest in making data Findable, Accessible, Interoperable, and Reusable (FAIR) has often resulted in centralized platforms. However, when data governance and sovereignty structures do not allow the movement of data, other methods, such as federation, must be pursued. Implementation of fully federated data approaches are not without their challenges. The user experience may become more complicated, and federated analysis of unstructured data types remains challenging. Advancement in federated data sharing should be accompanied by improvement in federated learning methodologies so that federated data sharing becomes functionally equivalent to direct access to record level data. In this article, we discuss federated data sharing approaches implemented by three data platforms in the ADRD field: Dementia’s Platform UK (DPUK) in 2014, the Global Alzheimer’s Association Interactive Network (GAAIN) in 2012, and the Alzheimer’s Disease Data Initiative (ADDI) in 2020. We conclude by addressing open questions that the research community needs to solve together.

## Introduction

Science is a data-driven economy. Access to high-quality data is the *sine qua non* of creating knowledge and deriving benefit. Data sharing mandates from journals and funders are now requiring studies to make data accessible (Nature Methods, 2023). However, in the health sciences, data access is challenging. For example, in a survey of 3,556 articles from 333 open access biomedical journals, only 7% of corresponding authors responded positively to a data access request, even when their intention to share data was explicitly stated. In this experiment, the revealed preference of 93% of authors was to not share (Gabelica et al., 2022).

For the Alzheimer’s disease and related dementias (ADRD) community, these challenges have been recognized for some time, resulting in the development of several data sharing platforms. The first of these was the Laboratory of Neuro Imaging (LONI) (Rex et al., 2003), which supported many data sharing projects including Alzheimer’s Disease Neuroimaging Initiative (ADNI) (Mueller et al., 2005) in the Image and Data Archive (IDA) network (Crawford et al., 2015). However, data relevant to the ADRD community goes beyond imaging to include a broad mix of population and clinical cohort data, genetics, experimental medicine data, and randomized trials data. These study designs cover a range of data modalities varying in scale, complexity, and sensitivity, including –omics, imaging, and electronic health records. Platforms for different data modalities include CPAD for trials data, NACC and ALZ-NET for clinical data, NIAGADS for genetic data and FinGen for electronic health record data (Table 1). These datasets also vary in governance requirements with some being freely available (open access), others requiring specific permissions (restricted access) and some only being available to the data controller (closed access). More recently multi-modal, multi-cohort repository and analysis platforms have developed to reflect the complexity of Alzheimer’s disease. These include the Global Alzheimer’s Association Interactive Network (GAAIN) (Neu et al., 2016), the Dementias Platform UK (DPUK) (Bauermeister et al., 2020), and the Alzheimer’s Disease Data Initiative (ADDI) (Alzheimer’s disease Workbench, 2020). Collaboration between these initiatives has made it apparent that a more general data sharing infrastructure is required to simplify and streamline data access across platforms.

**TABLE 1:** Summary of dementia related data platforms.

Database	URL	Description	Datasets	Subjects	Reach	Access	Functionality				
							Data discovery	Data analysis	Data federation	Data transfer	Data model
The Global Alzheimer’s Association Interactive Network	https://gaaindata.org/	AD-related data platform, federated access to ∼*n* = 500,000 data	61	533,218	Global	Platform application	✓	✓	✓	X	X
Alzheimer’s Disease Neuroimaging Initiative	https://ida.loni.usc.edu/login.jsp	Centralized clinical and imaging data from 23 studies	3	3,208	Global	Cohort application	✓	X	X	X	X
Alzheimer’s Disease Data Initiative	https://www.alzheimersdata.org/	Centralized clinical and imaging data from 48 studies, enables workspace analysis	48	ongoing	Global	Platform application	✓	✓	✓	✓	C-Surv
Dementias Platform UK	https://www.dementiasplatform.uk/	Centralized clinical and imaging data from 60 studies, enables workspace analysis	60	3.6 m	Global	Platform application	✓	✓	✓	X	C-Surv
The National Institute on Aging Genetics of Alzheimer’s Disease Data Storage	https://www.niagads.org/	Genetic data	95	172,701	Global	Cohort application	✓	X	X	X	RS number
National Alzheimer’s Coordinating Center	https://naccdata.org/	Centralized ADRC data	1	45,923	United States	Platform application	✓	X	X	✓	X
Critical Path for Alzheimer’s Disease	https://c-path.org/programs/cpad/	Clinical Trial data from industry	41	6,500	United States	Platform application	✓	X	X	✓	X
Alzheimer’s Network Alzheimer’s Association	https://www.alz-net.org/	Real world clinical and imaging data	ongoing	ongoing	Global	Application	✓	✓	X	X	X
Alzheimer’s Disease Knowledge Portal	https://adknowledgeportal.synapse.org/	Centralized access to data, provides workspace	12	n/a	Global	Application	X	X	X	X	X
FinnGen	https://www.finngen.fi/en	Centralized data repository	1	589,000	Finland	Application	✓	X	X	X	X
The EU Joint Programme Neurodegenerative Disease Research	https://neurodegenerationresearch.eu/	AD-related worldwide data platform	175	120,000	Global	Cohort application	✓	X	✓	X	X
Integrative analysis of Longitudinal Studies on Aging	https://www.maelstrom-research.org/network/ialsa	Centralized dataset search platform	25	70,000	Global	Cohort application	✓	X	X	X	X
European Progression of Neurological Disease	http://europond.eu/	Models and tool platform	n/a	n/a	Europe	Tool development	X	X	X	X	X
NeuGrid	https://www.neugrid2.eu/	Models and tool platform	n/a	n/a	Europe	Tool development	X	X	X	X	X
European Platform for Neurodegenerative Diseases	https://epnd.org/	AD-related worldwide data platform,	60	120,000	Europe	Platform application	✓	X	X	X	X
Dementias Platform Australia	https://www.dementiasplatform.com.au/	Centralized clinical and imaging data	ongoing	ongoing	Global	Platform application	✓	✓	✓	X	C-Surv
Dementias Platform Korea	https://kdrc.re.kr/eng/about/vision.aspx	Centralized clinical and imaging data	ongoing	ongoing	Republic of Korea	Platform application	✓	✓	✓	X	X
Alzheimer’s Disease Data Viewer	https://adata.scai.fraunhofer.de/	Centralized dataset search platform	20	72,372	Global	Platform application	✓	X	X	X	Multiple

Current solutions are constrained by (i) increasingly complicated data sharing requirements with barriers stemming from institutional, ethical, or legal obligations, (ii) a trend toward bespoke institution-specific platforms that are not designed for data sharing across institutions, and (iii) variability in workflows for the same research question across platforms that can introduce unwanted variation into the findings. These barriers pose significant challenges for data sharing and collaboration. GAAIN, DPUK and ADDI are actively developing a set of innovative solutions that enable data access at scale and pace across platforms to alleviate some of the barriers. Specifically, we offer solutions that:

(1) Resolve the complex pattern of the stakeholder involvement by providing streamlined data sharing agreements designed for use with multi-lateral collaborations.
(2) Provide decentralized data sharing solutions that can operate globally across platforms whether they be institute-specific or institute-agnostic.
(3) Establish universally accessible analysis using workspaces and containerized software that allow the use of standard workflows across platforms.

**FIGURE 1:**
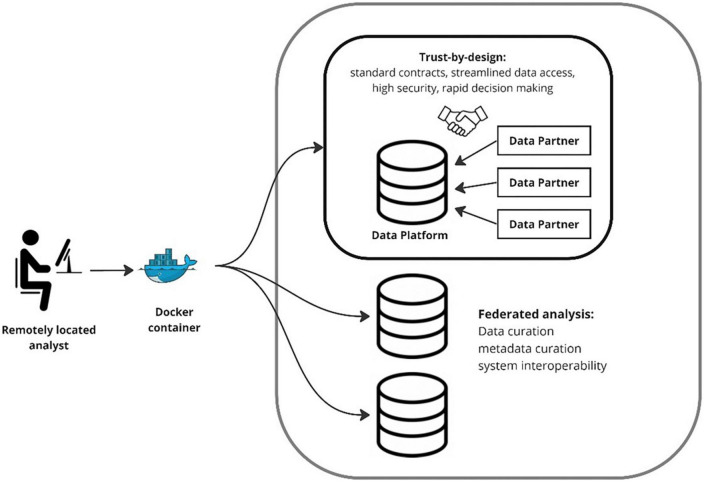
Trust by design and data federation.

The two key design principles that underpin delivery of these solutions by these platforms are trust-by-design and data federation (Figure 1).

### Trust-by-design

Trust-by-design involves a shift away from bespoke bilateral agreements toward trust in a system. Trust is the implicit operating principle underlying collaborative science, simplifying otherwise complex and unpredictable environments, identifying points of certainty around which to organize, and agreeing on a culture where key uncertainties are removed on the basis of mutual agreement, respect, and ethical codes. By facilitating better prediction of the likely reciprocal behavior of others in sharing costs and benefits, trust fosters collaboration. Trust is also foundational to the provenance of data and technologies. As datasets grow in size, complexity, and sensitivity, confidence in the provenance chain becomes increasingly central to the viability of an analysis. Although informal trust-based solutions work well for bilateral collaboration, emerging research questions frequently require multi-lateral collaboration involving large numbers of diverse stakeholders. Multiple actors with multiple interests involving multiple data-sources generate complexity leading to potentially prohibitive transaction costs. Trust-by-design solutions provide the information necessary for accurate and rapid judgments of trustworthiness and scientific value. Here, legal, privacy, security and scientific requirements are embedded within technical and organizational workflows that are explicit, transparent, and fully auditable. This enables systematic streamlining, standardization, and automation. It is trust in systems that underpins federated analysis at scale and pace.

### Data federation

Data federation is a mechanism allowing researchers to remotely query a dataset residing at source without ever seeing record-level entries. Within the ADRD community, the data assets that are available are a mix of open, restricted, and closed repositories, with more sensitive data generally being held in restricted or closed environments. Federation requires research questions to be formed into a computation task to be submitted, where the results returned from the submitted task(s), subject to disclosure control where applicable. This pragmatic solution allows data contributors to share datasets that would have otherwise remained inaccessible due to data transfer being undesirable or prohibited due to governance constraints, or unfeasible due to scale. Limitations in federation include data discovery, the wide range of variables, and the diversity of data models used across datasets. Addressing these requires high levels of forward planning and coordination. Although these limitations can be mitigated by high quality metadata and the use of standard data models, they remain a challenge for most federated analysis. Limitations apart, by adopting pragmatic strategies and respecting local legal, consent, privacy, and compute concerns, data federation is an increasingly used analysis strategy.

This article describes trust-by-design and federation solutions implemented by GAAIN, DPUK, and ADDI. We conclude by addressing open questions that the research community needs to solve together and by inviting others to join the data sharing movement.

## The Global Alzheimer’s Association Interactive Network (GAAIN)

### Background

Building on the pioneering work of LONI and learnings from ADNI and over 100 other multi-site, multi-modality, cross-sectional and longitudinal studies, GAAIN was the first platform to facilitate data discovery, access, and analysis for ADRD research data. Whilst LONI and ADNI offered centralized imaging data storage, and access (upon approval) to researchers around the world, GAAIN extended the notion of collaborative research in ADRD to a federation model where data can be accessed remotely while preserving data ownership and local, distributed archives. GAAIN addressed concerns of the scientific community regarding data ownership by allowing disease-related data stored in independently operated repositories to be accessed remotely. This enabled data partners to maintain data ownership while providing federated access to users with minimal disruption to the data owners’ systems. Since its inception in 2012, the breadth and depth of data accessible through GAAIN has grown to host more than 60 data partners and 500,000 subjects’ data from around the world.

### Data utilities

Global Alzheimer’s Association Interactive Network is optimized for federated analysis and supports exploratory analysis prior to the submission of a formal access request. By bringing together data discovery tools and contact details, GAAIN simplifies the selection of, and access to, datasets. Distinctive features include:

(1) Federated data access and processing wherein data and data repositories of the different data partners stay within their respective infrastructure.
(2) A secure platform of data sharing that is not disruptive to the data partners’ systems.
(3) Protection of the policies and ownership of the data.
(4) Directly coupled data exploration and analytics within GAAIN, enabling multi-subject, multi-project, and multi-institutional data aggregation.
(5) Integration of brain imaging metrics via execution of federated processing pipelines.
(6) Federated access to other data platforms that can be connected to DPUK and ADDI.
(7) Harmonization between variables that allows pooling and analysis of different data sets.

### Informatics architecture

The GAAIN system architecture comprises a central server that communicates with multiple client applications (Data Partner Clients or DPCs) that are installed in the data partner sites. The DPC is a Java jar file that contains both a light-weight webserver and database (H2 database) that does not disrupt existing systems. This allows GAAIN to remotely connect to data partners without ever having the data stored centrally (federated) unless the data partner decides to do so. The DPC allows remote access to tabular data and brain imaging data. Upon connection to the different DPCs, the data partners appear in the web interface and investigators can explore the available data.

The GAAIN Interrogator (GAAIN, 2017) is the main infrastructure by which investigators can inspect and interact with data in GAAIN. It differs from other data browsing interfaces by allowing dynamic data exploration and visualization through the definition of cohorts. Charts and line graphs make selections visually intuitive where the investigator can choose characteristics from a search range and thus dynamically adjust the cohort definitions. Users can also conduct analysis on the browser using the available data and cohort definitions.

The cohorts of interest can be further used to initiate brain pipelines (for example, executing a brain volumetry analysis on subjects with a certain MMSE range). These brain pipelines are executed remotely on the data partner’s site via the DPC in the form of a containerized software (Docker). The results of these pipelines are returned to the interrogator as new variables and can be further analyzed.

Each data partner has complete ownership and control of the data, and data transfer is not required. The data partner signs a non-legal binding Memorandum of Understanding (MOU) before joining GAAIN that formalizes GAAIN’s data sharing policies and other terms and conditions of GAAIN participation. Data partners have complete control over data access and display and can disable their DPC at any time thus removing connection to GAAIN.

### Summary

Global Alzheimer’s Association Interactive Network’s data sharing policies and systems are tailored to provide an intuitive and voluntary integration of multiple Alzheimer’s disease data repositories within a common sharing network. Combining data from different data partners requires infrastructure like that implemented in GAAIN but also appropriate ontologies to enable cross-cohort search and data aggregation.

## Dementias Platform UK (DPUK)

### Background

At inception, the DPUK Data Portal was designed to facilitate access to UK population and clinical cohort data. It has since developed to provide an end-to-end data management service for cohorts, clinical studies, trials, and systematic reviews. Currently it facilitates access to 60 cohorts representing individual-level data for 3.6 million participants.

### Data utilities

The DPUK Data portal is optimized for multi-modal pooled analysis enabling epidemiologic, imaging, genetic, proteomic, and clinical data to be combined. However, it also has federated analysis capability. Distinctive features include:

(1) Curation of data to research readiness using common standards according to modality (Bauermeister et al., 2023).
(2) A suite of data discovery tools.
(3) Centralized management of access requests.
(4) Personal analysis space with a wide range of standard and specialist software packages.
(5) Data hubs for specialist research groups and consortia.
(6) Synthetic data for preliminary model testing.
(7) Federated access to other data platforms including GAAIN, ADDI, DPAU (Dementias Platform Australia), and Korea Dementia Research Center (Korea Dementia Research Center [KDRC]).

### Informatics architecture

The Data Portal operates within the UK Secure eResearch Platform (SeRP, 2006) environment according to ISO 27001 (SeRP, 2006) as a data processor according to General Data Protection Regulation [GDPR] (2016) and Legislation.gov.uk (2018). Data may be accessed remotely for *in situ* analyses but not downloaded to third-party sites. Data-use approval remains with the cohort research teams who retain control over data access. Preparing datasets for third-party researchers and providing suitable documentation is resource intensive.

### Summary

The preparing of datasets by data contributors for third-party researchers is resource intensive. The Data Portal reduces this burden through the management of access requests on behalf of cohort research teams, the use of a common data model, and the development of standard documentation for data stored within the Data Portal. This obviates the need for repeated data transfer and pre-processing. The UKSeRP environment has been designed for use with linked electronic health records and is a suitable environment for the onward sharing of linked data.

## Alzheimer’s Disease Data Initiative (ADDI)

### Background

Alzheimer’s Disease Data Initiative’s mission is to accelerate AD research by enabling collaborative data sharing and analysis. ADDI’s trust-by-design solution is the Alzheimer’s disease (AD) Workbench (Alzheimer’s disease Workbench, 2020). The AD Workbench delivers access to key datasets around the world across public and private sectors using a secure cloud-based data platform. The AD Workbench provides data contributors with flexible data sharing options that preserve their branding and maintains their control and autonomy over the data through configurable data access requests and approval workflows. Along with storing some datasets locally, ADDI has achieved interoperability with DPUK, EPND (Bose et al., 2022), GAAIN and Vivli (Vivli, 2013). To make federated solutions accessible, ADDI has developed the Federated Data Sharing Appliance (FDSA), an option that offers both data providers and researchers a streamlined interface to access, maintain and query data where it resides.

### Data utilities

The AD Workbench is optimized for federated analysis. Distinctive features include:

(1) The FDSA is agnostic to data type. Currently querying is available on structural data.
(2) The FDSA is a stand-alone Linux application installable on local data provider’s IT environment and deployable on any infrastructure.
(3) Data contributors determine the level of permissioned access to the record-level data that is granted to researchers.
(4) The administrative module gives data contributors a point-and-click interface from which they can manage researcher access, data contributors:
  • Can review and approve Data Access Requests from users.
  • Have visibility of all research query tasks and task status.
  • Can verify, after execution, that the results do not include record-level data.
(5) Submission (upon approval) of container-based analyses across multiple platforms.

### Informatics architecture

The federated dataset must be stored as a PostgreSQL database. FDSA seamlessly connects to the datasets. Docker is used to execute user-submitted tasks. FDSA includes a GUI Administrative module running on an onboard web server and a back-end service that manages and executes admin and end user tasks. A common set of research APIs can be used to access the data. FDSA requires minimal infrastructure for installation: 2 CPUs, 8GB of memory, and 100GB of storage.

### Summary

With this solution, ADDI has enabled sharing for data contributors who previously were unable to make their data available to researchers. FDSA is installable on-premise and is suited for a diverse range of datasets, data contributor, and data consumer needs. Under active development, FDSA will continue to add features, such as (1) trusted containers that do not necessitate manual review from data contributors, (2) a secure way for FDSA instances to communicate for combined analyses of federated datasets, and (3) a way for users to share models and analyses via containers with the community. ADDI’s federated solution removes another barrier to permissioned data access and further enables the research community to make novel discoveries by unlocking access to previously unreachable datasets.

## The wider environment

There are many variations on the theme of data sharing and any attempt to compile a comprehensive list will certainly be incomplete. Nonetheless, we endeavored to summarize Alzheimer’s-related data initiatives and identify similarities and differences to support analysts in considering which platforms are most relevant to their research question (Table 1). Overall, platforms follow a centralized model with various access tiers (open, restricted, closed) and high-level data discovery tools. Few platforms, however, provide data curation/metadata curation or access to computing resources.

## The way ahead

These platforms have several commonalities. They are working together to support the FAIR principles of data management (Findable, Accessible, Interoperable and Reusable) (Wilkinson et al., 2016), and are working toward the Dublin Core metadata specification (Dublin Core, 1994). For federated computation, all platforms support the GA4GH Task Execution Standard (GA4GH, 2013) as a suitable candidate for the containerization of analyses. Their collaboration also allows for the automated creation of containers to support standard analyses. Nevertheless, each platform provides distinctive data access options, recognizing that insisting on a single data platform for all use cases would stifle innovation, whilst agreement on commonly used standards facilitates collaboration. The use of federation alongside standardized analysis can further render the data reusable with researchers continuously accessing and processing the data from multiple studies in a similar way. This can have tremendous impact on ADRD scientific discovery where previously unseen relationships can now emerge via a unified access and analysis model (Neu et al., 2017).

However, federated data sharing is not without challenges. Further integration across platforms is focused on widening the availability of data for federated approaches and work has begun on a framework where datasets from each of the three platforms can be discovered from within the others. GAAIN datasets can be discovered from ADDI and, upon approval, data can be transferred to the AD Workbench. Datasets from DPUK are requestable from the AD Workbench for access at federated level. Handling of multimodal data is a key challenge for a comprehensive federated model. Integration of clinical, neuroimaging and genetics data is essential. To give researchers access to multimodal data, GAAIN has made efforts toward this direction by establishing external connections to the IDA network (Crawford et al., 2015) that hosts a variety of studies. This effort can be augmented by ADDI’s AD Workbench tools that are agnostic to any data format. In addition, DPUK has already established ontologies and multi-modal analysis that can be further integrated with ADDI’s products. Key to progress is increasing cross-platform interoperability through data standards, efficient data access, and distribution of computational workload.

Implementing data standards across platforms would be transformative. A common ontology (data model) alleviates the data preparation burden for researchers and developers. A recent study comparing data preparation times for 25 variables in two cohorts found that using the ‘bespoke’ cohort designed data model required 5–6 h per cohort, whilst using a standard data model reduced this time to 30 min per cohort (Bauermeister et al., 2023). Standard ontologies also simplify the building of data discovery tools for developers, as standard metadata models enable tools such as data dictionaries to have broader application across datasets and be harmonized across platforms. However, data standards require consensus, and this will vary according to data modality. GAAIN, DPUK, and ADDI are working together to identify, develop, and test potential data models according to data modality. For example, the ontology implemented by DPUK can be integrated with GAAIN and ADDI.

Analyses conducted in federated settings pose unique opportunities and challenges for data access. Federated approaches increase the potential base of data enabling the design of purpose-specific cohorts, i.e., using existing data to create cohorts designed to address specific research questions. An example of this is the GAAIN Interrogator tool (GAAIN, 2017), a web-based application that allows users to query and explore distributed datasets related to Alzheimer’s disease and other neurodegenerative disorders. These cohorts can be characterized using persistent unique identifiers enabling rapid replication and re-purposing. A challenge, however, is the efficient running of models across diverse datasets and informatics architectures. A solution under development within the consortium is the creation of synthetic datasets (Muniz-terrera et al., 2021) that model the characteristics of the original data. These can be used to develop task-specific ‘boilerplate’ code that is known to operate successfully across platforms and to test the functionality of models across platforms prior to a formal analysis. For higher-order data (imaging and genomics) this approach is time and computationally efficient. By running and verifying models on simulated data, researchers can spend less time submitting federated queries and data providers may have fewer queries to review.

The federated analysis framework has increasing potential for federated learning applications. In federated learning, different data partners/clients train their neural networks, and a central model aggregates the parameters of that model (Rieke et al., 2020). This approach allows the training of large-scale neural network models without the need to access centralized data (Stripelis et al., 2022).

We hope in the future to release a skeleton of the underlying federated analysis framework from these platforms. By doing so, outside researchers can build their own federated methods and models and those can potentially be integrated with the proposed platforms.

Computational workload becomes an increasingly important resource constraint as the scale of datasets grows with a commensurate increase in the complexity of research questions. For federated analyses there is a requirement to establish models of ‘smart’ federation where requests and computational load can be efficiently managed. Computational and labor-intensive burdens on data providers lead to bottlenecks and longer turnaround times to review and fulfill data access requests. Additionally, extracting information from large cohorts of interest requires increased computational resources. GAAIN, DPUK and ADDI provide distinctive solutions to this challenge, each based around the functionality of its trust-by-design architecture. ADDI’s AD Workbench cohort information can be utilized within a user’s workspace to create analysis only for this cohort. To increase efficiency and transparency, requests and datasets need to be in specific format before computational resources are allocated. GAAIN is also working on identifying a specific format for how these requests can be more efficient in terms of how they allocate resources. In DPUK, the analysis plan determines the computational resource that is allocated to the project. All platforms are working toward modality specific formats that can be integrated within docker containers.

A further challenge is the scrutiny of findings to ensure that data or personally identifiable information is not observed or downloaded. Safeguards that prevent this from happening can include presentation of summary statistics rather than single data points or preventing analysis of cohorts consisting of few subjects (Neu et al., 2016). Currently this is an arduous task on all platforms; a solution that does not scale and is vulnerable to error. Machine learning provides a potential solution for automating the management of data leakage risk (Shabtai et al., 2012).

## Data availability statement

The original contributions presented in this study are included in the article/supplementary material, further inquiries can be directed to the corresponding authors.

## Author contributions

AT, MP, and JG contributed to conceptualization and initial draft of the manuscript. All authors contributed to the writing, reviewing, and editing and approved the submitted version.
